# A Clinical Study of Toxication Caused by Carbamazepine Abuse in Adolescents

**DOI:** 10.1155/2018/3201203

**Published:** 2018-03-22

**Authors:** Wei Xu, Yu-Lin Chen, Ying Zhao, Li-Jie Wang, Jiu-Jun Li, Chun-Feng Liu

**Affiliations:** Department of Pediatrics, Shengjing Hospital of China Medical University, Shenyang, China

## Abstract

Carbamazepine is known to produce the side effect of euphoria. As such, it lends itself to being a drug of abuse, particularly in the adolescent population. This retrospective study evaluated carbamazepine abuse, treatment course, and associated morbidity in Chinese adolescents. The median dose of carbamazepine resulting in overdose was 2,000 mg (800–5,000). Patients were largely from urban-rural fringe areas (76.47%, 52.94%) with school performance within the last 1/3 range and (52.94%) unsupervised by parents. 35.29% experienced an obvious sense of euphoria. All patients had nervous system symptoms, 6 (35.29%) cases developed coma (GCS < 8), and 5 (29.41%) cases experienced convulsion. Four cases were treated with hemodialysis. The incidence rate in young patients with repeat carbamazepine use and without the supervision of parents was higher than that in first-time users (5/7 versus 4/10), but the difference was not significant. The toxic dose of repeat users was 3428 ± 1035 mg, significantly higher than that of 1470 ± 646 mg in first-time users (*P* = 0.001). Carbamazepine can produce a sense of euphoria, which is more likely to lead to its abuse and overdose in adolescents. To prevent carbamazepine abuse and overdose will be critical in educating at-risk adolescents and preventing associated morbidities in the future.

## 1. Introduction

Carbamazepine is often used for treatment of seizure disorders and neuropathic pain [1]. It is used off-label as a second-line treatment for bipolar disorder and in combination with an antipsychotic when treatment with a conventional antipsychotic alone has failed [2]. Carbamazepine, as a tricyclic drug, can produce a sensation of euphoria. This is generally noted as a side effect of long-term use, as seen in individual reports [3, 4]. However, experts have vigilantly pointed out in some case reports that its abuse potential may be brought about by the euphoric effect of the drug [4, 5].

Substance use in adolescents is an important public health concern. Previous research [5, 6] has identified adolescence as the peak period for initiation of substance use, an epidemic which imparts large health burdens in this age group. Substance use often progresses to abuse, and in adolescents this may involve tobacco, alcohol, and/or illicit drugs (e.g., cannabis, amphetamine-type stimulants, cocaine, opioids, and novel psychoactive substances or so-called legal highs). It is well accepted that tobacco and alcohol abuse occurs across all continents and countries to varying extents. While drug abuse is frequently reported in the countries of South Africa, America, and Northern Europe [5], there are few reports in other areas of the world, perhaps due to lack of data or relevant studies.

Substance use in Chinese adolescents is rarely reported. Moreover, little is known about carbamazepine use in adolescents who have progressed to abuse and/or overdose. Thus, the objective of the present study was to characterize patterns of carbamazepine use and resulting morbidities in a population of adolescents admitted to the pediatric intensive care unit of Shengjing Hospital of China Medical University. To our knowledge, this is the first such study of carbamazepine abuse and overdose. We analyzed and summarized social data along with clinical management of these patients and wish this study could attract the concerns about carbamazepine abuse from all social circles and provide some help for the clinical treatment of patients with carbamazepine toxication.

### 1.1. Patients

A retrospective review was conducted of all available adolescent patients admitted to the pediatric intensive care unit with carbamazepine overdose between January 2015 and July 2016 at Shengjing Hospital, China Medical University. This study was approved by the Medicine Ethics Committee of Shengjing Hospital of the China Medical University (2016PS288K). Clinical data were collected in a manner that maintained patient privacy; patient records and information were anonymized and deidentified prior to analysis.

Diagnostic criteria [7] of toxication included (1) a history of carbamazepine use; (2) carbamazepine concentration in venous blood ≥ 12 *μ*g/ml as tested after admission; (3) clinical findings of change of consciousness, altered mental status, or limb movement disorders; and/or (4) respiratory depression. There were no exclusion criteria.

## 2. Methods

We ensure that our manuscript reporting adheres to the STROBE guidelines for the reporting of this observational study.

Blood carbamazepine concentration was tested using the TDxFLx System (Abbott, USA) in the standard fashion. Within two hours after hospital admission, two ml of venous blood was collected from each patient. The test was completed within two hours after the serum was separated.

A data collection form was used to obtain information from the patients' legal guardians including birth history, personal growth and development history, parent marital and economic status, guardian status, school location, school performance, and past medication history of patients. Patients were asked about their reason for carbamazepine use, feelings after use of the drug, and medication status of similar age neighborhood peers. Other clinical data were collected by medical staff.

A database was established using Excel software (2007) with double entry of the following clinical data: age, gender, home address, course of disease, guardian status, history of substance use, concomitant medication, blood drug concentration, main laboratory tests, course of treatment, and prognosis.

### 2.1. Statistical Analysis

Data analysis was conducted using the SPSS 13.0 software. Discrete variables were expressed as counts (percentages), and continuous variables were expressed as means ± standard deviation (SD). Differences in the demographic and clinical characteristics of patient groups were assessed using the chi-square test for categorical variables. Continuous variables were analyzed using Student's *t*-test; *P* ≤ 0.05 was considered statistically significant.

## 3. Results

### 3.1. Demographics

Table 1 displays descriptive characteristics of the study. The patients (see supplementary Table S1) ranged in age from 12 to 14 years with the majority of individuals being female (58.82%) and from urban-rural fringe areas (82.35%). All patients were students of grades 6-7, with 52.94% (*n* = 9) having school performance at the 33rd percentile or less. In all, 52.94% (*n* = 9) patients were not supervised by their parents more than one year. Of these, five were living with their grandparents due to their parents being divorced (*n* = 3) or working in foreign areas (*n* = 2). Additionally lack of parental supervision was due to enrollment in boarding school (*n* = 3) and there was one case in which the patient's father was imprisoned and their mother was lost.

### 3.2. Substance Use

All patients were admitted to the hospital for carbamazepine overdose. The median dose ingested was 2,000 mg (range 800–5,000 mg) with a drug dose ≤ 1,500 mg in 4 cases. The lowest carbamazepine blood level detected within six hours of admission was 15.34 *μ*g/ml. The majority of patients (70.59%) had carbamazepine blood levels greater than 20 *μ*g/ml (upper limit monitored in our hospital). All 17 cases were treated by a full gastric lavage within 1.5 hours after ingestion of carbamazepine. Self-reported reasons for carbamazepine use included being a “cool thing” associated with personal loyalty (41.18%), a funny thing (23.53%), a sense of euphoria after repeated use (23.53%), and lack of desire to attend school (11.76%). Of all patients, 41.18% used the medication two or more times. With regard to other factors of importance, 35.29% experienced a conscious sense of euphoria, 11.76% used methadone, 11.76% drank coca cola, 5.88% had a history of alcohol abuse, and 5.88% (*n* = 1) used methadone and drank coca cola. All patients had an average of 4 classmates (range 2–10 classmates) with carbamazepine abuse (dose: 200–1,500 mg).

### 3.3. Clinical Manifestations

Clinical manifestations of carbamazepine overdose occurred 1.94 ± 1.14 hours after ingestion. The median time for hospital admission due to overdose was 6 hours (range 2–24 hours) after ingestion of carbamazepine. Frequently encountered clinical manifestations were dizziness and headache (100%), disequilibrium (88.24%), vomiting (76.47%), syncope (76.47%), and conscious disturbance (64.71%). All eleven patients found to have conscious disturbance underwent MRI examination. Five of these patients were found to have white matter edema and the remaining cases were unremarkable. Less frequent sequelae noted as a result of carbamazepine overdose included Glasgow Coma score (GCS) < 8 (35.29%), aspiration pneumonia (35.29%), convulsions (29.41%), coma and skin abrasion (23.53%), and coma and convulsion (11.76%). Paroxysmal hypopnea during coma occurred in four cases (23.53%) but there were no cases of respiratory failure. Fever was noted in 23.53% of patients, as well as urinary and fecal incontinence in 17.65% of patients. In addition, 35.29% had mild arrhythmias (sinus bradycardia (*n* = 3), sinus tachycardia (*n* = 1), and multiple premature ventricular contractions (*n* = 2)) but no circulatory dysfunction. At one hour after admission, 64.71% had leukocytosis > 10 × 10^9^/L with a dominant elevation of neutrophils. Elevated blood glucose > 7 mmol/L was noted in 35.29% (*n* = 6) of the patient population. Four patients (23.53%) experienced mild hypokalemia and one (5.88%) experienced mild hyponatremia. Pathologic alterations in blood levels of C-reactive protein, procalcitonin, hemoglobin, platelets, creatinine, and aminotransferases were not found in any patients.

### 3.4. Treatment and Prognosis

All 17 patients were treated with full gastric lavage within six hours of admission. Of the nine patients found to have coma (GCS < 8) or convulsion, four were treated with 2-3 cycles of hemodialysis. The remaining five cases did not receive such treatment due to personal reasons. There was no significant difference noted in the length of coma, number of convulsions, length of stay, or prognosis, in patients treated with hemodialysis when compared with those who did not undergo hemodialysis. Other treatment included catharsis, fluid replacement, and diuresis. All patients were discharged with full recovery.

### 3.5. Carbamazepine Use Patterns

As is shown in Table 1, patients were divided into two groups: first-time carbamazepine users and repeat carbamazepine users; their demographic factors and clinical course were assessed within each group. There was no significant difference between these two groups with regard to gender, school performance in the 33rd percentile or less, concomitant medication, a sense of euphoria, GCS < 8, or convulsion. More frequent carbamazepine abuse was observed in patients without parental supervision more than one year (*P* ≈ 0.05). At the time of overdose, repeat carbamazepine users had higher toxic doses than first-time carbamazepine users, and this difference was significant (*P* = 0.01). There was no significant difference in the time to admission between the two groups.

## 4. Discussion

In China, substance use in adolescents is frequently reported, and similarly to the majority of countries most of these reports focus on tobacco and alcohol [8, 9]. Alcohol abuse is markedly elevated [10] in Chinese left-behind children, as these children lack parental guardianship and are ultimately at risk for adverse behaviors and suicide [11]. In contrast to left-behind children, most Chinese teenagers living in urban areas are the only child in their families and due to strict family management are infrequently involved with substance abuse. Due to economic factors, adolescents residing in rural areas are less likely to experience drugs abuse when compared with those that reside in urban areas. At present, there is a paucity of literature specific to carbamazepine abuse and addiction. In this study, most patients with carbamazepine abuse were from urban-rural fringe areas and lacked parental supervision, thus permitting access to and abuse of drugs. Carbamazepine is a prescription drug that should not be easily accessed by adolescents. However, in some areas it can be conveniently bought from drugstores without certification or limitation to the quantity purchased. Clearly, such loose management opens the door to the potential for substance use and abuse.

Along with other national data about adolescent and teenage behavior, trends in risky behavior may be gleaned by surveillance through poison centers. With over 5,000 annual reports to the poison centers about intentional exposures on school property, school personnel, parents, and guardians must be cognizant of pharmaceutical and nonpharmaceutical substances used for abuse, misuse, or suicide [12]. This study describes demographics and use patterns of adolescents admitted for carbamazepine overdose. Importantly, none of the patients in this study had a history of suicidal ideation. A carbamazepine intoxication with suicide attempt is a relatively common clinical problem in adult, which could lead to death [13]. The mortality rate due to carbamazepine toxicity is around 13% [14]. Indeed, children in this study all showed an active attitude to life—their substance use was attributed to inadequate care or psychological immaturity without full recognition of the toxicity and side effects of drugs, as demonstrated by self-reported reasons for use of carbamazepine. Reasons for carbamazepine use were not liking to attend school in two patients and feeling that learning was boring or poor school performance in 13 patients. Thus, more care for these children was needed from the schools, the guardians, and the society to help them understand and enjoy learning, such that they had a productive school life. The study showed when children have a sense of euphoria after one dosing, they are more likely to take multiple doses. Based on the data in this study from first-time users, the incidence rate of euphoria was relatively low. However, the concomitant use of methadone or coca cola could overlap the central excitation effect of carbamazepine, thus facilitating the production of a sense of euphoria [15].

Children of 12 or 13 years of age are in a rapid stage of developing cognition and cannot accurately judge social phenomena; they are known to have strong curiosity and an urgent need to be accepted by the society. In this framework, their tendency toward substance use to attain happiness or to make more friends is understood. In other studies of drug abuse and overdose in adolescents, the guardianship of parents played a crucial role in behavior restriction, cognitive education, and psychological health [16, 17]. Of the repeat users in the present study (*n* = 7), five were without parental guardianship more than one year. Without question, this lack of guardianship was detrimental to their psychological heath and recognition of drug hazards, to the point where they believed such harmful freedom of action might provide them with a sense of safety. Our analysis showed that the percentage of children without parental guardianship was far higher than the demographic data in the local areas. Moreover, poor school performance was more outstanding in this age group, and the majority of children had school performance at the middle to low levels. This brings into question whether the education neglect of parents contributes to the occurrence of mental disorders or a low awareness of dangers in children.

In this study, patients generally presented with apparent clinical manifestations within one to two hours after ingestion, and then they were rapidly sent to the hospital by their classmates or teachers who had a wary awareness after finding the toxication symptoms. Different from suicidal poisoning or accidental poisoning, the major clinical manifestation of patients was the nervous system symptoms after timely gastric lavage and other effective treatment. Respiratory depression was seen in a single patient for whom mechanical ventilation-assisted respiration was not required. Arrhythmias were common and not malignant; severe arrhythmia and circulatory disorders were not observed. Six cases were diagnosed as aspiration pneumonitis. Of those, two cases presented with significant fever and cough productive of sputum. The remaining four cases presented with crackles in the lungs and had inflammatory changes in chest imaging. Therefore, aspiration pneumonitis could not be distinguished from pulmonary edema caused by carbamazepine toxicity as reported [18]. Chronic carbamazepine toxicity is noted by decrease in circulating leukocytes, while acute toxicity is related to a marked increase of leukocytes [19]. However, it is unclear whether the stress reaction or other mechanisms are involved. In the present study, no patients had pathologic levels of liver enzymes, myocardial enzymes, or serum creatinine, also differing from the clinical manifestations of toxicity in a study of 33 epileptic patients [20].

High-dose carbamazepine abuse (up to 5,000 mg) was noted in this study but there were no deaths. In human beings, the lethal dose of carbamazepine is not clarified yet. According to the American Food and Drug Administration [21], four cases of death induced by carbamazepine overdose have been reported. Two cases were with adults at a dose of 3.2 g (a 24-year-old woman died of a cardiac arrest and a 24-year-old man died of pneumonia and hypoxic encephalopathy) and two cases were with children at 4 g (a 14-year-old girl died of a cardiac arrest) and 1.6 g (a 3-year-old girl died of aspiration pneumonia). The result of no death in our study might be because toxicity was found in a timely manner, and all patients were treated by an active, full gastric lavage and other measures to promote toxicant excretion within six hours after medication ingestion. As for blood purification treatment, plasmapheresis or hemodialysis was used in case reports [22, 23]. In this study, four patients were treated by plasmapheresis for severe coma or convulsion, but five patients refused blood purification treatment due to personal reasons. Clinically, there were no significant differences in the total treatment time and prognosis between these two groups. Therefore, whether blood purification is required in carbamazepine overdose needs to be further studied.

Our study is the first report of carbamazepine abuse and addiction in Chinese adolescents. Carbamazepine abuse in Chinese adolescents older than 14 years is unknown yet as in China, the patients older than 14 years need to seek medical care in the adult departments. The mechanism of carbamazepine abuse should be further addressed as this was a limitation of the present study. Additionally, the relevant social, mental, educational, and euphoric factors should be deeply investigated. Moreover, an epidemiological study of carbamazepine abuse in adolescents is also needed. However, in a view of carbamazepine abuse, addiction, and overdose, the responses to substance use in adolescents will differ substantially depending on their age, stage of life, level of substance use, and their socioenvironmental context. In thinking about the responses to substance use in adolescents, one must first take into account the differences to the adult population that adolescents experience during this period of rapid growth and development. These include the rapid physiological development during puberty, which can affect cognitive reasoning, emotional regulation, and risk taking [6, 24]. To address this issue requires increased societal engagement, reasonable guardianship of parents, proper education from schools, and the advocacy of pediatricians. Of course, the identification and medical care capacity of toxicity resulting from substance use are the most fundamental guarantee for the life safety of the concerned children.

## 5. Conclusion

Carbamazepine can produce a sense of euphoria, which is more likely to lead to its abuse and toxicity in adolescents who are living in the urban-rural fringe areas, have poor school performance, and are not supervised by parents. The main manifestation of carbamazepine toxicity is the nervous system symptoms, and the prognosis is good after active treatment based on timely detection. Strengthening drug management and adding relevant education for adolescents are suggested. In fact, the pediatricians and government shall take bigger responsibilities for propaganda and education about the damage of carbamazepine and other drugs to child health and the prevention of substance use in children [25]. In China, there is a long way for such work: at present, pediatricians lack resources to devote themselves to the demographic studies and large-sized epidemiological surveys, and no sufficiently effective channels are available to publicize the damage and seriousness of substance use at a professional level.

## Figures and Tables

**Table 1:** Demographic factors and clinical course for first-time and repeat users of carbamazepine.

	First-time users (*n* = 10)	Repeat users (*n* = 7)	*P*
Gender (male, %)	4 (40%)	3 (42.86%)	0.646
School performance within the last 1/3 range (*n*, %)	4 (40%)	5 (71.43%)	0.218
No supervision of parents (*n*, %)	4 (40%)	5 (71.43%)	0.052
Concomitant medication (*n*, %)	2 (20%)	3 (42.86%)	0.314
Toxic dose (mean, mg)	1470 ± 646	3428 ± 1035	0.001
Sense of euphoria (*n*, %)	2 (20%)	4 (57.14%)	0.145
Time to admission (mean, *h*)	6.5 ± 4.19	10.71 ± 9.63	0.310
GCS < 8 (*n*, %)	3 (30%)	3 (42.86%)	0.484
Convulsion (*n*, %)	3 (30%)	2 (28.57%)	0.686

## Data Availability

Most raw data of this study has been showed in Table 1; some of them were hidden due to the privacy policy. If anyone needs the whole database they could have access to it through email of Dr. Wei Xu: tomxu.123@163.com. But most of the data are in Chinese.

## Supplementary Materials

Supplementary Materials Supplemental Table 1: main characteristics of all 17 patients.
