# Supplementary material for: A Clinical Study of Toxication Caused by Carbamazepine Abuse in Adolescents
Source: Biomed Res Int. 2018 Mar 22;2018:3201203. doi: 10.1155/2018/3201203 (PMC5885346; doi:10.1155/2018/3201203)
Supplement: Supplementary Materials — Supplemental Table 1: main characteristics of all 17 patients. [file 3201203.f1.doc]

**Supplemental table 1. Main characteristics of all 17 patients.**

| No. | Gender | Age(Y) | School Performence | Parental Supervision | Toxic dose (mean, mg) | Concomitant medication | Times of medication | Number of medication friends | Sense of euphoria | Time to onset of clinical manifestations(h) | Time to admission(h) | Blood carbamazepine levels(μg/ml) | GCS <8 | Convulsion | Aspiration pneumonia | Arrhythmia | Respiratory depression | Hemoperfusion |
| --- | --- | --- | --- | --- | --- | --- | --- | --- | --- | --- | --- | --- | --- | --- | --- | --- | --- | --- |
| 1 | M | 13 | Middle 1/3 | Yes | 2000 |  | 1 | 5 |  | 3 | 6 | >20.00 | 7 |  | Yes | Yes |  |  |
| 2 | F | 13 | Last 1/3 | No | 4500 |  | 3 | 10 | Yes | 2 | 2 | >20.00 | 6 |  | Yes |  | Yes |  |
| 3 | F | 13 | Middle 1/3 | Yes | 2900 | methadone | 1 | 4 |  | 1 | 3 | >20.00 |  | Yes | Yes |  |  | Yes |
| 4 | M | 12 | Last 1/3 | No | 3400 | alcohol | 3 | 8 | Yes | 0.5 | 1.5 | 15.34 |  |  |  | Yes |  |  |
| 5 | F | 13 | Middle 1/3 | Yes | 1400 |  | 1 | 5 |  | 2.5 | 4 | >20.00 |  |  | Yes |  |  |  |
| 6 | F | 12 | First 1/3 | Yes | 900 |  | 1 | 2 |  | 1 | 2 | >20.00 |  | Yes |  |  |  |  |
| 7 | M | 12 | Middle 1/3 | Yes | 900 |  | 1 | 4 | Yes | 5 | 9 | >20.00 |  | Yes |  |  |  |  |
| 8 | M | 13 | Last 1/3 | No | 1000 |  | 1 | 3 |  | 3 | 5 | 19.86 |  |  |  | Yes |  |  |
| 9 | M | 12 | First 1/3 | Yes | 800 |  | 1 | 3 |  | 1.5 | 2 | 16.07 |  |  |  |  |  |  |
| 10 | M | 13 | Last 1/3 | Yes | 5000 | methadone | 4 | 7 | Yes | 2.5 | 3 | >20.00 | 7 | Yes | Yes | Yes | Yes |  |
| 11 | F | 13 | Middle 1/3 | No | 3000 |  | 2 | 6 |  | 2.5 | 4 | >20.00 |  |  |  |  |  | Yes |
| 12 | F | 14 | First 1/3 | Yes | 3500 |  | 3 | 5 |  | 0.5 | 2 | >20.00 |  |  |  | Yes |  | Yes |
| 13 | F | 13 | Last 1/3 | No | 1500 | coca cola | 1 | 4 |  | 2 | 6 | 19.68 |  |  |  |  |  |  |
| 14 | F | 13 | Last 1/3 | Yes | 1800 |  | 1 | 2 | Yes | 1.5 | 4 | >20.00 | 6 |  | Yes |  | Yes | Yes |
| 15 | F | 13 | Last 1/3 | Yes | 1500 |  | 1 | 3 |  | 2 | 5 | 19.46 | 7 |  |  |  |  |  |
| 16 | M | 13 | Last 1/3 | No | 2100 |  | 2 | 5 |  | 2 | 4 | >20.00 |  |  |  | Yes |  |  |
| 17 | F | 13 | Last 1/3 | No | 2500 | methadone coca cola | 3 | 2 | Yes | 0.5 | 3 | >20.00 | 6 | Yes |  |  | Yes |  |
